# Oral–Gut Microbiome Crosstalk in Cancer

**DOI:** 10.3390/cancers15133396

**Published:** 2023-06-28

**Authors:** Kentaro Inamura

**Affiliations:** Division of Pathology, The Cancer Institute, Japanese Foundation for Cancer Research, Tokyo 135-8550, Japan; kentaro.inamura@jfcr.or.jp; Tel.: +81-3-3570-0111

## 1. Introduction

Increased research efforts have led to a growing body of evidence on the human microbiota and its critical role in balanced health. The microbiota is involved in a variety of physiological processes, including metabolism, nerve transmission, blood circulation, and the immune response. An imbalance in microbial homeostasis, known as dysbiosis, is associated with a wide range of health disorders, including obesity, malnutrition, neurological disorders, behavioral disorders, and cancer [1]. The human microbiota is a complex, diverse, and abundant population of symbiotic microorganisms, which inhabit many sites in the human body, including the skin, mouth, and gut [2]. The mouth and gut, anatomically connected by the gastrointestinal tract, are the two largest microbial habitats in the human body. In healthy conditions, physical and chemical barriers (e.g., gastric and bile acids) segregate the mouth from the gut. However, in the absence of these barriers, the oral microbiota can translocate to the intestines and modulate the gut microbiota, potentially contributing to the pathogenesis of gastroenterological diseases and cancer. Furthermore, as with its involvement in Alzheimer’s disease, diabetes mellitus, and rheumatoid arthritis [3], the oral microbiota may systemically contribute to carcinogenesis. Conversely, the gut microbiota can be transmitted to the oral cavity via the fecal–oral route, potentially inducing gastroenterological disorders. To date, the large majority of studies have independently focused on these microbiomes in an organ-specific context, giving limited attention to the interplay between the oral and gut microbiomes. There is emerging evidence on the involvement of microbiomes in interorgan networks, such as the gut–brain axis [4] and gut–lung axis [5]; thus, the oral–gut microbiome axis and its involvement in disease development have recently attracted attention. In 2023, a large-scale metagenomic study has identified the person-to-person transmission landscape of the gut and oral microbiomes [6]. Gut microbiome transmission from mother to infant was substantial during infancy, and the transmitted microbes remained persistent even at older ages [6]. Conversely, the oral microbiome was primarily transmitted horizontally and was enhanced by the duration of cohabitation [6]. It was shown that 13% and 38% of the gut and oral microbial strains were shared by partners, respectively, indicating that microbes can horizontally spread across cohabiting individuals even in adulthood [6]. These findings highlight a non-negligible effect of social interactions in shaping the microbiome and suggest a potential contribution of the oral–gut microbiome axis to increasing the communicability of microbiome-associated cancers.

## 2. The Study by Park S.Y. et al. Published in *Cancers*

In their review article [7], Park et al. nicely highlighted the oral–gut microbiome interactions involved in the pathogenesis of gastroenterological diseases and cancer, including inflammatory bowel disease (IBD), colorectal cancer (CRC), liver diseases and cancer, and pancreatic cancer.

### 2.1. Inflammatory Bowel Disease (IBD)

IBD is a group of chronic inflammatory disorders, such as Crohn’s disease and ulcerative colitis, that affect the small and large intestines. Although oral microbes cannot inhabit the gut of healthy individuals due to the intact mucosal barrier, they can colonize the gut of IBD patients due to gut leakiness and barrier disruption. For example, *Fusobacterium nucleatum*, prevalent in the oral cavity but uncommon in the intestines of healthy individuals, frequently colonizes the gut of IBD patients. In mice with dextran sulfate sodium-induced colitis, orally administered *F. nucleatum* exacerbated colitis by damaging the intestinal barrier and inducing aberrant inflammation [8]. In mice with gut dysbiosis, *Klebsiella* species from the salivary microbiota colonized the gut and promoted T helper 1 cell induction and severe intestinal inflammation [9], as is often observed in IBD patients. These study results suggest that the oral microbiota contributes to IBD pathogenesis by colonizing the gut and causing dysbiosis and inflammation.

### 2.2. Colorectal Cancer (CRC)

As with IBD, patients with CRC suffer from intestinal barrier dysfunction, which allows oral microbes to colonize the gut. Oral microorganisms, such as *Fusobacterium*, *Parvimonas*, and *Peptostreptococcus*, have been found in the intestines of CRC patients, suggesting the involvement of the oral–gut microbiome axis in colorectal carcinogenesis [10]. *F. nucleatum*, an oral microbe, translocates to the gut of CRC patients, where this microbe appears to generate an immunosuppressive microenvironment by interacting with the host’s immune cells [11].

An in vitro study showed that *Porphyromonas gingivalis*, a key pathogen involved in periodontitis and known to aggregate and coinfect with *F. nucleatum*, invaded CRC cells and promoted proliferation of the infected CRC cells [12]. Consistent with this study, periodontitis and serum antibodies against *P. gingivalis* have been associated with increased CRC mortality [13]. Further, a meta-analysis showed that periodontitis is a potential risk factor for CRC development [14]. Overall, these studies indicate that the oral microbiome and its dysbiosis are associated with the occurrence and consequence of CRC via oral–gut microbial interactions.

### 2.3. Liver Diseases and Cancer

The oral–gut microbiome axis is being recognized as a key modulator in chronic liver disorders. Notably, metagenomic research has shown that the intestines of cirrhotic patients were invaded and colonized by oral microbes, most of which were found to be of buccal origin [15]. In addition, oral dysbiosis has been associated with chronic liver disorders: odontogenic infection by *P. gingivalis* was shown to promote the progression of non-alcoholic steatohepatitis by accelerating fibrosis through the activation of hepatic stellate cells [16].

Compared to healthy individuals, patients with hepatocellular carcinoma (HCC) have an altered oral microbiome: the salivary microbiota is less diverse in patients with liver diseases, including HCC. Moreover, the salivary microbiota of HCC patients was enriched with *Haemophilus*, *Porphyromonas*, and *Filifactor* species [17].

### 2.4. Pancreatic Cancer

The oral microbiome and its dysbiosis are associated with pancreatic carcinogenesis, as indicated by a meta-analysis that showed an association of periodontitis and edentulism with the incidence of pancreatic cancer [18]. In pancreatic cancer mouse models, oral administration of *P. gingivalis* accelerated the development of pancreatic ductal adenocarcinoma and promoted epithelial–mesenchymal transition, which was suppressed by probiotic supplementation [19]. *P. gingivalis* survived inside the pancreatic cancer cells, and the intracellular *P. gingivalis* boosted the tumor cell proliferation to the extent depending on the duration of its intracellular persistence [20].

Patients with pancreatic cancer exhibit oral dysbiosis; consequently, the signature of the oral microbiota may serve as a valuable diagnostic tool for detecting pancreatic cancer at its earliest stages. Indeed, metagenomic classifiers based on the gut and oral microbiomes have been shown to accurately identify patients with pancreatic cancer in different populations [21].

## 3. Conclusions

Despite numerous attempts to elucidate the involvement of microbiomes in tumor initiation and progression, most research has focused on an organ-specific microbiome. Given the emerging evidence indicating the involvement of microbiomes in interorgan networks and the person-to-person transmission of the gut and oral microbiomes, in-depth research is needed to examine the oral–gut microbiome network implicated in cancer development and progression. Along with the gut microbiome, the oral microbiome could serve as a promising diagnostic or prognostic tool for a multitude of cancer types and could be exploited as a therapeutic target. Furthermore, modulation of the oral microbiota by improved dental hygiene or probiotic supplementation is a promising strategy for controlling microbiome-associated cancer.

## Abbreviations

CRC: colorectal cancer; HCC: hepatocellular carcinoma; IBD: inflammatory bowel disease.
